# Comparing the use of mathematical calculation to a measuring wheel to determine distance walked in three different course configurations of the 6 minute Walk Test in healthy adults

**DOI:** 10.1080/07853890.2022.2041209

**Published:** 2022-02-16

**Authors:** Suzanne K. O’Neal, Megan C. Eikenberry, Alexander Bocchi, Kyle Carroll, Michelle Fettig, Parker Folliard, Clara Martinez

**Affiliations:** Doctor of Physical Therapy Program, Midwestern University, Glendale, AZ, USA

**Keywords:** Walking capacity, walking test, gait, outcome measures

## Abstract

**Introduction:**

A recent clinical practice guideline set forth recommendations for the administration of the 6 Minute Walk Test, including course set-up and using mathematical calculation to obtain the distance walked. In clinical practice and research, however, deviations from these protocols exist.

**Purpose:**

To assess for differences in total distance walked between use of mathematical calculation and a measuring wheel during three different course configurations of the 6 Minute Walk Test.

**Methods:**

Fifty healthy adults (18 males, 32 females) completed this study. The mean age was 37.04 (13.76) years ranging from 23 to 61 years. Each participant completed three course configurations of the 6 Minute Walk Test: a 12-meter straight walkway representing the Academy of Neurologic Physical Therapy Core Set of Outcome Measures Clinical Practice Guideline protocol, a 30-meter straight walkway, representing the American Thoracic Society’s recommended protocol, and a 1.2-meter by 12-meter rectangular walkway, of which the Core Set of Outcome Measures Clinical Practice Guideline was derived. For mathematical calculation, the total number of laps counted, and this total number was multiplied by the distance of one lap with any partial lap added. Additionally, a research assistant followed behind each participant with a measuring wheel to capture distance walked.

**Results:**

For all configurations, there were statistically significant differences between mathematical calculation and a measuring wheel, with mathematical calculation producing significantly less total distance. Additionally, there were statistically significant differences between all course configurations, despite the method of measurement.

**Conclusion:**

Adhering to 6 Minute Walk Test protocols, including the method of measuring the distance, is imperative to accurately interpret results and compare to existing data.

**Key messages**
- Despite recommendations for standardized administration of the 6 Minute Walk Test, deviations exist, including the method of which to obtain the total distance walked; either by use of mathematical calculation or a measuring wheel.
- In three different 6 minute walk test course configurations, including the American Thoracic Society’s recommended protocol and the Academy of Neurologic Physical Therapy recommended protocol, the measuring wheel resulted in significantly larger distances than use of the mathematical calculation.
- Despite the measuring wheel able to account for the turns during the 6 Minute Walk Test, it is imperative for clinicians to utilize standardized procedures such as using mathematical calculation, in order to accurately track progress and compare to existing data, of which mathematical calculation was used to derive.

## Introduction

The 6 Minute Walk Test (6MWT) is an outcome measure originally developed as a submaximal test of functional capacity for patients undergoing treatment for moderate to severe heart or lung disease [1]. Since then, the test has also been used to assess walking ability in people with various neurologic conditions, such as Parkinson disease [2], spinal cord injuries [3], Alzheimer disease [4], and stroke [5]. The American Thoracic Society (ATS) attempted to standardize the 6MWT by publishing guidelines, which included specifics on test set-up and administration [1]. In 2014, the European Respiratory Society (ERS) and ATS created an ad hoc task force to review field walking tests, including the 6MWT, and develop technical standards [6]. Per these standards, a straight course of 30 meters or more in length should be used, with the ends of the course clearly marked [6]. The person undergoing the test walks back and forth around each cone as many times as possible in six minutes, while the assessor keeps a tally of the number of laps the person completes. When completed, mathematical calculation is used to determine the total distance. Despite the ATS and subsequently the ERS/ATS task force attempting to standardize test administration, there have been deviations from this protocol. One systematic review looked at 6MWT protocol variations in people with stroke [7]. Out of 127 studies assessed, only 67 (52.8%) provided specific description of the walkway used, and out of these, only 18 (26.9%) used the ATS recommended 30-meter walkway. The rest used shorter lengths (*n* = 10), longer lengths (*n* = 26) or continuous walkways in the shape of an oval, square, or rectangle (*n* = 13). Of the straight walkways, the distance ranged from 10 meters to 150 meters [7]. This review highlighted the significant variability in the set-up of the 6MWT.

The Academy of Neurologic Physical Therapy (ANPT) attempted to further standardize the administration of certain outcome measures with the creation of the Core Outcome Measures work group who published the Core Outcome Measures Clinical Practice Guideline (COM CPG) [8]. The COM CPG included a new, standardized protocol for the 6MWT, adapted from a course used in a study by Quinn et al. [9]. In this study, a 12-meter walkway was used, with a two-cones wide width of 124 centimeters (1.24 meters), therefore allowing the participants to walk in a rectangular fashion. The COM CPG modified this by recommending a straight 12-meter walkway with one cone at each end and a walkway width of 124 centimeters to allow for turning, thus participants make a narrower trajectory [8]. Of note is that this 12-meter length is significantly shorter than the ATS guidelines, therefore may be more feasible in smaller healthcare settings. The COM CPG protocol also instructed the clinician to multiply the number of laps by 12 and add the distance of any partial lap to obtain the total distance [8]. Despite the use of mathematical calculation being the standard protocol for both the original ATS protocol as well as the current COM CPG protocol, deviations exist in the method of measurement used in both clinical practice and research, with some clinicians and researchers utilizing a measuring wheel [10,11]. It has been shown that the distance of the 6MWT walkway as well as the shape of the walkway can significantly influence the distance walked [12–15]. It has also been shown that the use of a measuring wheel compared to the conventional method of calculating the number of laps for the total distance walked can result in significant differences in healthy children and adolescents [16]. To date, no studies have compared the use of mathematical calculation to use of a measuring wheel during the 6MWT in the healthy adult population. Additionally, there is a lack of studies exploring the newly recommended COM CPG course or comparing it to the original ATS standardization. The purpose of this study was to compare two methods of measurements during three different course configurations of the 6MWT in the healthy adult population, including the newly established COM CPG course configuration. The researchers hypothesized that there would be statistically significant differences between the two methods of measurement in all three course configurations of the 6MWT.

## Materials and methods

### Study design

This was a cross-sectional, prospective study. Prior to data collection, this study was approved by the Midwestern University Institutional Review Board (AZ 1298) and supported by an intramural grant provided by Midwestern University (no grant number established by the University).

### Role of the funding source

The funders played no role in the design, conduct, or reporting of this study.

### Participants

Convenience sampling was used to recruit potential participants from the local area. Inclusion criteria were healthy adults aged 18 to 65 who had the ability to walk at least 30 min at a leisurely pace in six-minute intervals. Participants were excluded for any of the following reasons: chest pain during exercise, current use of beta-blockers and/or calcium channel blockers, any current condition affecting their gait or balance, any current active or chronic conditions which compromised their cardiovascular or respiratory capabilities, any reported cognitive impairment, resting systolic blood pressure >165 mmHg and/or a diastolic blood pressure >110 mmHg, and/or a resting heart rate >70% of age-predicted maximum heart rate.

### Sample size

An a priori sample size estimation was conducted using G*Power, version 3.1 by a Midwestern University Biostatistician. The calculation was based on an alpha of 0.05, power of 0.80, and an effect size of 0.50. It was estimated that a minimum of 44 participants were needed for this study. Fifty participants were recruited to account for any dropouts or exclusions.

### Data collection

**Figure 1.**
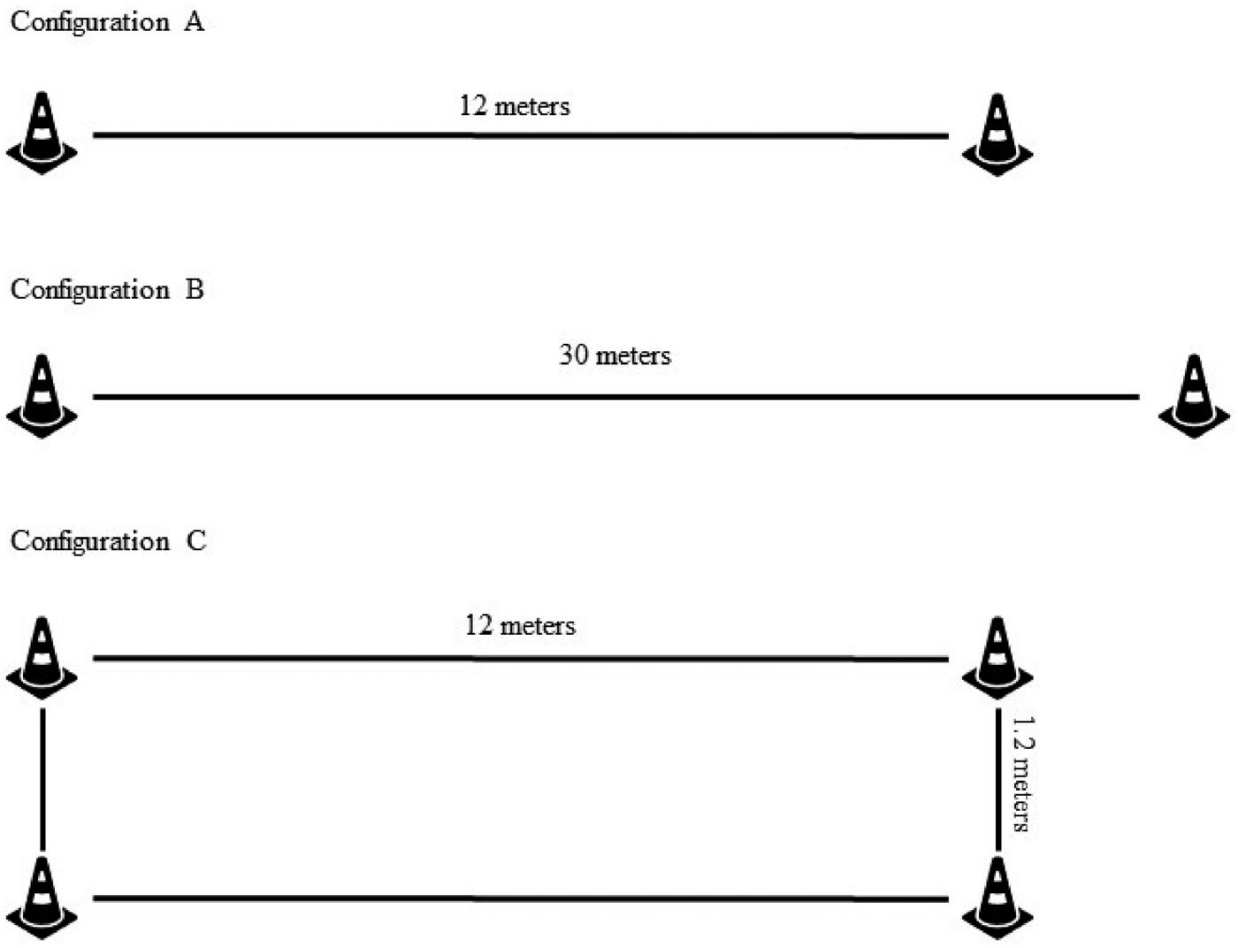
Course layout for the three configurations. Configuration A represents the ANPT OM CPG recommended course, configuration B represents the ATS recommended course, and configuration C represents a course layout from which configuration A was adapted.

Each participant was scheduled a single data collection session and performed one trial of each of the three configurations during this session. Five second-year students enrolled in the Doctor of Physical Therapy program at Midwestern University assisted with data collection. Each research assistant (RA) was trained on the format of the study and given a document which included the standardized instruction script to be read to each participant (Table 1). Prior to data collection, all aspects of the study was provided to each participant and written informed consent was obtained, including consent that de-identified data from the study may be shared with the research community at large to advance science and health. Demographics were collected, including age and gender. Testing was performed in a large gymnasium with even, solid, smooth flooring. Three course configurations were set-up within this gymnasium (Figure 1). Configuration A, representing the ANPT COM CPG recommendations, was a 12-meter straight path with one cone placed at each end [8]. Configuration B, representing the ATS protocol, was a 30-meter straight path with one cone placed at each end [1,6]. Configuration C, representing a course configuration of which the COM CPG guidelines were derived, was a rectangular path of 12 meters in length and 1.2 meters in width, with one cone placed at each corner of the rectangular path [9]. Three participants were scheduled at the same time and each started on one of the configurations. An RA was present at each configuration to instruct and supervise the participant. In attempts to minimize bias, the principal investigator and co-investigator were not involved in the collection or recording of data.

**Figure 2.**
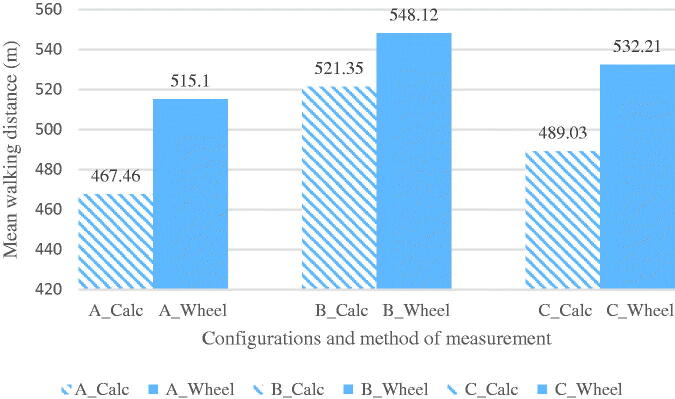
Mean walking distance comparison between methods of measurements during three 6 Minute Walk Test configurations. *Note*: each comparison was a statistically significant difference (*p*<.001). A = 12-meter straight walkway; B = 30-meter straight walkway; C = 1.2-meter by 12-meter rectangular walkway; Calc: mathematical calculation; Wheel: measuring wheel.

**Table 1.** Standardized instructions.

The aim of this test is to walk as far as possible for 6 min. You will walk back and forth in the pattern described to you prior. Six minutes is a long time to walk, so you will be exerting yourself. You may get out of breath or become tired. You are allowed to slow down, to stop, and to rest as necessary. You may come to a complete stop but resume walking as soon as you are able. You are not permitted to sit during a rest break. If you must sit, the test will be over. Do you understand the instructions? I am going to follow behind you with this measuring wheel to track your distance. Remember the aim is to walk as far as possible in 6 min, but do not run or jog. Start now or when you are ready.

### Reliability

Prior to data collection, reliability was established for each method of measurement.

To establish reliability for this study, five assessors and nine participants were used. Three participants came in simultaneously and were randomly assigned a testing sequence (ABC, CAB, or BCA). The participants performed each configuration twice, each with a different assessor. Prior to each test, pre-vitals were taken, including blood pressure, heart rate, and oxygen saturation. During the test, the assessor followed behind the participant with a measuring wheel and a smartphone application which contained a lap counter and a stopwatch. At the end of each test, the assessor calculated the distance walked both by mathematical calculation based on the number of laps completed, and by the distance measured by the measuring wheel. After each test, the participant rested until vitals returned to within 10% of baseline. The participants returned one week later to complete each of the 6MWT configurations, each with one of the same assessors previously assigned to that configuration. For statistical analysis, the intraclass coefficient (ICC) was used. A two-way random-effect model based on single ratings and absolute agreement was used to assess inter- and intra-rater reliability. Mean estimations along with 95% confidence intervals (CI) were reported for each ICC. Values were interpreted as follows: >0.90 = excellent, 0.75 − 0.90 = good, 0.50 − 0.75 = moderate, <0.50 = poor [17].

For configuration A (12-meter walkway), the inter-rater reliability was excellent for both the calculated measurement (ICC = 0.92, [0.44–0.98]) and measuring wheel (ICC = 0.94, [0.70–0.99]), while intra-rater reliability was good for the calculated measurement (ICC = 0.86, [0.44–0.97]) and excellent for the measuring wheel (ICC = 0.95, [0.78–0.99]). For configuration B (30-meter walkway), the inter-rater reliability was excellent for the calculated measurement (ICC = 0.97, [0.85–0.99]) and measuring wheel (ICC = 0.99, [0.93–1.00]) with excellent intra-rater reliability for the calculated distance (ICC = 0.92, [0.63–0.99]) and measuring wheel (ICC = 0.96, [0.83–0.99]). For configuration C (12 m by 1.2 m walkway), the inter-rater reliability was excellent for the calculated distance (ICC = 0.95, [0.79–0.99]) and measuring wheel (ICC = 0.98, [0.93–1.00]), and the intra-rater reliability was excellent for the calculated distance (ICC = 0.95, [0.75–0.99]) and measuring wheel (ICC = 0.96, [0.84–0.99]).

### Procedure

After written informed consent was obtained, each participant was randomly given one of three cards which indicated the order in which they would perform the three course configurations (ABC, CAB, or BCA). The participant was seated in a chair and baseline vital signs were taken including blood pressure, heart rate, and oxygen saturation. The RA then demonstrated the correct path to take for the configuration and read a set of standardized instructions (Table 1). During the test, the RA followed behind the participant as to not influence the walking pattern or speed and used a measuring wheel in one hand to trace the participant’s walking path. The measuring wheel (Komelon USA) used in this study had a single, 4ʺ diameter wheel with an adjustable handle and a gear driven distance counter with a push button reset. The measuring wheel can measure distances up to 10,000 feet and had a reported accuracy within 0.2%, or ± 2 inches in 100 feet in ideal conditions. Ideal conditions were defined by the manufacturer as a straight, flat, and smooth surface. To measure distance, the RA placed the wheel on the ground, and followed the participant, pushing the wheel within the path. The RA ensured that contact was being made to the ground at all times, while the gear wheel kept track of the distance by advancing the counter. The RA also used a lap counter smartphone application to keep track of the number of laps completed. During testing, the RA did not talk to the participant except to alert the participant when each minute had elapsed, with some brief encouragement. For example, “You are doing good, you have five more minutes”. After six minutes, the participant was instructed to stop. If the participant stopped at any point between the cones, this position was marked, and the partial lap distance was measured with a measuring wheel. The participant was seated back in the chair and vital signs were taken immediately, and every two minutes until they returned to within 10% of baseline. Once this was achieved, the participant was escorted to the next configuration, as indicated on their testing order card. These procedures remained the same for each configuration. To calculate the total distance walked by mathematical calculation, the number of full laps completed was multiplied by the total distance of one full lap. Any partial lap distance was added to this total for the total distanced walked. To obtain the measuring wheel distance, the distance from the measuring wheel counter was recorded.

### Data analysis

Data were analyzed using IBM SPSS Statistics for Windows, Version 24.0 (IBM Corp., Armonk, NY, USA). Normal distribution was determined using the Shapiro–Wilk test. A paired *t* test was run to determine differences between the two methods of measurements in each course configuration. To compare the three course configurations, a repeated measures ANOVA was used and post hoc tests with Bonferroni correction were run to locate the source of difference, if significant. Effect size estimates were reported using Cohen’s *d* and interpreted based on guidelines proposed by Cohen with 0.20 to <0.50 = small effect; 0.50 to <0.80 = medium effect; and >0.80 = large effect [18].

## Results

Fifty healthy participants (18 males, 32 females) completed this study. The mean age was 37.04 (13.76) years ranging from 23 to 61 years. All participants were able to complete the three 6MWT configurations in its entirety. In between tests, most participants needed less than two minutes of rest, with a range of less than two minutes to six minutes.

Figure 2 shows the mean result of each course configuration and each method of measurement. A paired *t* test revealed significant differences between the methods of measurement in each configuration (Table 2). When comparing the course configurations using mathematical calculation, a repeated measures ANOVA F(2, 98) = 71.95, *p*<.001 revealed statistically significant differences in distances walked between the configurations at an alpha .05 level. When comparing course configurations using a measuring wheel, a repeated measures ANOVA F(2, 98) = 32.44, *p*<.001 revealed statistically significant differences in distances walked between the configurations at an alpha 0.05 level. Table 3 shows the pairwise comparisons of each configuration with each method of measurement.

**Table 2.** Mean difference between two methods of measurements during three 6MWT course configurations.

Configuration	Method of measurement	Mean distance (m)	Mean difference (m)	*p*	Effect size (*d*)
A	Calculation	467.46 (67.89)	−47.64 (14.02)	<.001*	0.70
	Wheel	515.10 69.16)			
B	Calculation	521.35 (72.88)	−26.77 (8.01)	<.001*	0.37
	Wheel	548.12 (73.46)			
C	Calculation	489.03 (65.25)	−43.18 (15.47)	<.001*	0.64
	Wheel	532.21 (70.63)			

*Statistically significant difference, *p*<.001.

*A* = 12-meter straight walkway.

*B* = 30-meter straight walkway.

*C* = 1.2-meter by 12-meter rectangular walkway.

**Table 3.** Pairwise comparisons of three 6MWT course configurations using two different methods of measurements.

Comparisons	Mean difference (m)	*p*	Effect size (*d*)
Calculation			
A – B	−53.89 (4.90)	<.001*	0.77
B – C	32.32 (4.19)	<.001*	0.47
A – C	−21.57 (4.45)	<.001*	0.32
Wheel			
A – B	−33.02 (4.52)	<.001*	0.46
B – C	15.91 (3.41)	<.001*	0.22
A – C	−17.11 (4.29)	.001*	0.24

*Statistically significant change, *p*<.05.

*A* = 12-meter straight walkway.

*B* = 30-meter straight walkway.

*C* = 1.2-meter by 12-meter rectangular walkway.

## Discussion

The 6MWT is commonly used in clinics to assess walking capacity, and psychometric data have been reported for a variety of patient populations, including stroke, spinal cord injury, Alzheimer disease, total hip arthroplasty, and older adults [3,4,19–22]. However, in order to accurately compare to these data, adherence to standardized protocol is important. There have been attempts to standardize administration protocols by the guidelines set by the ATS, ERS/ATS task force, and the ANPT COM CPG, including the length and shape of the walkway, and the method of which to obtain the total walking distance. Studies have reported deviation from this protocol, including the course configuration and the method of measurement. A previously mentioned systematic review, which included 127 studies assessing the use of the 6MWT with people with stroke reported 26 different methods to which the 6MWT was administered [7]. A meta-analysis regarding the use of the 6MWT in people with multiple sclerosis also reported differences in protocol, including the method of measuring the distance [23], and other studies have used a measuring wheel within its study design [10,11]. This variability can make it difficult for clinicians to accurately interpret results and track patient progress.

In this present study, the results revealed that using mathematical calculation to obtain 6MWT results produced significantly less distance compared to the use of a measuring wheel in all three course configurations. Additionally, configuration A, which represents the ANPT COM CPG recommendation of a 12-meter straight walkway, produced the largest mean difference between the two methods of measurement, in which the calculated method produced a mean difference of −47.64 meters compared to the measuring wheel. This is most likely due to the higher number of 180-degree turns produced by this configuration. It has been shown that use of mathematical calculation is not able to account for the distance during the turns in the adolescent population [16]. Normative values for the mean age of our study are not available; however, this mean difference exceeds the minimal detectable change value for other populations, including chronic stroke [19], Alzheimer disease [4], and spinal cord injuries [3]. It also exceeds the minimal clinically important difference value in people with stroke with a slow gait speed of <0.40 m/s [5]. This is an important consideration as configuration A, a 12-meter straight walkway, represents the course recommended by the COM CPG, which is the most recent standardization guideline [8]. This walkway, compared to the ATS recommended walkway of 30-meters, is significantly shorter, therefore an increased number of 180-degree turns is required. The use of a measuring wheel consistently produced greater distance compared to mathematical calculation. Because of this, the use of a measuring wheel has the potential to result in incorrect test interpretation in which there is a perceived positive change. Additionally, the COM CPG protocol allows for a patient to be able to perform the 6MWT even if physical assistance is needed. The use of mathematical calculation, as indicated on the COM CPG protocol, would allow clinicians’ hands to be free in order to safety assist and guard the patient during the test.

When comparing the course configurations, there were significant differences between the three configurations, regardless of the method of measurement. Configuration A, representing the COM CPG recommended course, consistently produced significantly lesser distance than the other two courses. Additionally, the longer ATS course of 30 meters consistently produced the greatest distance walked compared to the other two courses. These results are consistent with previous studies which also reported that different lengths and shapes of the walkway produced different results, with the longer walkways and/or continuous walkways resulting in greater distances walked in people with various conditions [12,13,15]. Ng et al. (2011) also reported that the number of turns performed during the 6MWT is inversely proportional to the length of the walkway, therefore the longer the walkway, the less amount of turns needed [13]. The longer walkway and the decreased amount of turns can allow an individual to accelerate and maintain a steady speed for a greater amount of time compared to a shorter walkway, where acceleration and deceleration can require distances up to 5.08 metres total [13]. Given this statistic, requiring up to 5.08 metres of acceleration and deceleration would mean that up to 42.3% of a 12-meter walkway, as recommended by the COM CPG, would be needed for speeding up and slowing down before and after each turn. This further supports the need for updated psychometric data for this walkway length in order to have accurate data for comparison and patient tracking.

This data highlights the need to adhere to recommended standardization guidelines, as this is critical to ensure that any perceived positive or negative change on the 6MWT is actual change and not due to discrepancies in measurement methods or course configurations. This data showed that the two methods of measurement can produce significantly different results, therefore clinicians should be cognizant of this, and be mindful of adhering to established guidelines. A measuring wheel may be more accurate in capturing the actual distance walked during the 6MWT, however the error implicated in using this device is not yet known for adults in standardized 6MWT protocols. Additionally, with the use of a healthy population in our study, it also highlights the need for standardization beyond the neurologic population, as the COM CPG was intended.

### Study limitations

This study presents with several limitations. The mean age of our participants was 37 years, therefore the results cannot be generalized to older adults. The results can only be generalized to the healthy population. Additionally, each RA attempted to walk behind the participant and follow their path, however, there does exist the potential of deviation from the participants’ actual walking path. Mathematical calculation error potentials also exist, however we attempted to minimize risk of this error by having a second RA review and calculate the distance to ensure the results matched. Despite these limitations, this is the first study to the researchers’ knowledge which highlights the impact of the method of measurement on the distance walked during the 6MWT in the healthy adult population. It is also the first study to include the newly recommended course configuration included in the COM CPG.

### Conclusion

Adhering to the newly established protocol standards, including course configuration and method of measurement, is imperative to accurately report results and track changes. This study showed that the use of mathematical calculation versus a measuring wheel can produce significantly different results. This was true for three different course configurations, including a course recommended by the ATS and the course recommended by the COM CPG. Clinicians should adhere to set guidelines, which include use of mathematical calculation, as recommended by both the ATS guidelines and the COM CPG protocol, to remain consistent and be able to accurately compare to already established psychometric data.

## Data Availability

The data that support the findings of this study are openly available in Figshare at https://doi.org/10.6084/m9.figshare.18737438.v1
